# Lesions of the Intergeniculate Leaflet Lead to a Reorganization in Circadian Regulation and a Reversal in Masking Responses to Photic Stimuli in the Nile Grass Rat

**DOI:** 10.1371/journal.pone.0067387

**Published:** 2013-06-19

**Authors:** Andrew J. Gall, Laura Smale, Lily Yan, Antonio A. Nunez

**Affiliations:** Department of Psychology and Neuroscience Program, Michigan State University, East Lansing, Michigan, United States of America; Simon Fraser University, Canada

## Abstract

Light influences the daily patterning of behavior by entraining circadian rhythms and through its acute effects on activity levels (masking). Mechanisms of entrainment are quite similar across species, but masking can be very different. Specifically, in diurnal species, light generally increases locomotor activity (positive masking), and in nocturnal ones, it generally suppresses it (negative masking). The intergeniculate leaflet (IGL), a subdivision of the lateral geniculate complex, receives direct retinal input and is reciprocally connected with the primary circadian clock, the suprachiasmatic nucleus (SCN). Here, we evaluated the influence of the IGL on masking and the circadian system in a diurnal rodent, the Nile grass rat (*Arvicanthis niloticus*), by determining the effects of bilateral IGL lesions on general activity under different lighting conditions. To examine masking responses, light or dark pulses were delivered in the dark or light phase, respectively. Light pulses at Zeitgeber time (ZT) 14 increased activity in control animals but decreased it in animals with IGL lesions. Dark pulses had no effect on controls, but significantly increased activity in lesioned animals at ZT0. Lesions also significantly increased activity, primarily during the dark phase of a 12:12 light/dark cycle, and during the subjective night when animals were kept in constant conditions. Taken together, our results suggest that the IGL plays a vital role in the maintenance of both the species-typical masking responses to light, and the circadian contribution to diurnality in grass rats.

## Introduction

Circadian rhythms, which have a period of approximately 24 hours, are internally driven and can be entrained by daily light/dark (LD) cycles such that they adopt a period of exactly 24 hours, thus enabling animals to anticipate predictable daily changes in the environment. Photic cues can also contribute to rhythms by directly inhibiting or stimulating activity, a process referred to as masking [1]. Circadian rhythms are driven by the suprachiasmatic nucleus (SCN), a retinorecipient region of the hypothalamus that may also play a role in masking [2], [3]. The intergeniculate leaflet (IGL), which receives direct retinal input and is reciprocally connected with the SCN [4], [5], has also been implicated in regulating entrainment and masking [6], [7].

In nocturnal rodents, such as hamsters, mice, and rats, the IGL modulates entrainment of the circadian system to LD cycles as well as to some non-photic stimuli [8]–[15]. Specifically, IGL lesions alter the phase angle of entrainment and the rate of re-entrainment to phase shifts of the illumination cycle [9], [10], and can modify the period of free running rhythms in constant conditions as well as its responses to changes in light intensity [16], [17]. The IGL is also involved in dawn-dusk signaling in hamsters [18]. In addition, IGL lesions result in an enhanced negative masking response to light in hamsters, but not in rats [6], [11]. Therefore, there is evidence that the IGL is involved in both entrainment of circadian rhythms and masking by light in some nocturnal species.

In contrast to nocturnal species, very little is known about the role of the IGL in diurnal species. The only study examining the effects of IGL lesions in a diurnal mammal was conducted with *Octodon degus*. In that species, damage to the IGL led to a significant advance and lengthening of the active phase of the cycle under 12:12 LD conditions. These results could be accounted for by a change in masking, circadian regulation, or both [19]. However, masking responses to light and darkness following IGL lesions have not been examined in a diurnal species.

Here, we evaluated the hypothesis that the IGL mediates masking to photic stimuli in a diurnal rodent, the Nile grass rat (*Arvicanthis niloticus*). Since the IGL receives retinal input in both diurnal and nocturnal species [20]–[23], it represents a site where a common photic signal could be transduced differentially to support species-specific masking responses [1], [24]. Our approach used IGL lesions in grass rats and exposed them to acute 1-h dark and light pulses while monitoring their general activity. In addition, we recorded their activity under 12:12 LD conditions, constant darkness (DD), and constant light (LL). Our results suggest that in the diurnal grass rat, the IGL is not only involved in mediating species-specific masking responses, but that it also contributes to the circadian regulation of activity patterns in a phase-specific manner.

## Materials and Methods

All experiments were carried out in accordance with the National Institutes of Health Guide for the Care and Use of Laboratory Animals (NIH Publication No. 80-23) and were approved by the Institutional Animal Care and Use Committee of Michigan State University. All efforts were made to minimize the number of animals used.

### Subjects

A total of 42 adult female grass rats (*Arvicanthis niloticus)* were obtained from a breeding colony maintained at Michigan State University [25]. Virgin female grass rats in our colony do not show signs of estrous cycles with respect to vaginal cytology or changes in general activity (T. L. McElhinny & L. Smale, unpublished data). All animals in this study were singly housed in Plexiglas cages (34×28×17 cm). Infrared motion detectors (IRs; Visonic, Tel Aviv, Israel) were placed on top of each cage to monitor general locomotor activity in 5-min bins using the VitalView Program (Mini-Mitter, Bend, OR, USA). PVC pipe (length: 8 cm, diameter: 6 cm) was placed inside each cage to help reduce animals’ stress during cage changes, as described previously [24]. Food (PMI Nutrition Prolab RMH 2000, Brentwood, MO) and water were provided to animals *ad libitum*. Animals were either maintained in a 12:12 LD cycle (300 lux of white light during the light phase and complete darkness during the dark phase), in LL (300 lux of white light), or in DD (complete darkness). In 12:12 LD conditions, lights on occurred at ZT0. Grass rats were monitored for at least 2 weeks prior to surgery to determine their pattern of general activity in 12:12 LD conditions. Surgery was performed following this 2-week baseline-recording period. 34 animals received bilateral electrolytic lesions aimed at the IGL, and 24 of them survived (70.6% survival rate). 8 animals received sham surgery as controls (100% survival rate). Therefore, a total of 32 grass rats (*n* = 24 lesions, *n* = 8 shams) were included in the analyses presented here.

### Experimental Procedures

#### Surgery

Grass rats were anesthetized with isoflurane anesthesia and secured in a stereotaxic apparatus (Stoelting Co., Chicago, Illinois, USA). The top of each animal’s head was shaved, and then injected with a local anesthetic (Lidocaine, Hospira Inc., Lake Forest, IL; 6 mg/kg, s.c.). Artificial tears (Butler Company, Columbus, OH) were then applied over the eyes. An incision was made to open the scalp, four small holes were drilled in the skull, and the dura was punctured. Using an insulated tungsten microelectrode (A-M systems, Model 5770, 500 µm Diameter, 12 MOhm Impedance, Sequim, WA, USA), bilateral electrolytic lesions were made in 2 electrode placements (for a total of 4 lesions) aimed at the IGL using the following coordinates, with the tooth bar set at 0: AP: +0.05 and –0.01 cm from bregma, ML: ±0.28 cm from midline, and DV: –0.33 cm ventral to the meningeal surface. Lesions were made using a lesion-producing device (Stoelting Co., Chicago, Illinois, USA; Model #58040) to deliver 2.0 mA of DC current for 35 s. Autoclips were used to close the incision, and antiseptic ointment (Nolvasan Antiseptic Ointment, Fort Dodge, IA; 1% chlorhexidine acetate in 10% sterile alcohol base) was applied. The sham control group (*n* = 8) experienced the same procedure except current was not applied. To prevent dehydration, all animals received sterile saline (Abbott, s.c.; 1.0 mL). Immediately following surgery, all animals were administered an analgesic of ketoprofen (Fort Dodge Animal Health; s.c.; 5 mg/kg body weight). 24 and 48 h after surgery, all animals were given meloxicam (Boehringer Ingelheim Vetmedica Inc., St. Joseph, MO, USA; 0.2 mg/kg) delivered in a small piece of apple in order to reduce pain [26]. Animals recovered in their cages for at least 1-h on a warming blanket, and were immediately moved back to the recording room under standard 12:12 LD conditions and activity was continuously monitored. Autoclips were removed between 7–10 days after surgery. We used electrolytic lesions, instead of lesions that spare fibers of passage, because injections of excitotoxic agents aimed at the IGL cause extensive damage to nearby structures, such as the hippocampus [27]. In addition to damaging fibers of passage, electrolytic lesions can also cause retrograde damage to brain structures afferent to the targeted area.

#### Light treatment procedure

Following surgery, general activity was recorded for at least 4 weeks in standard 12:12 LD conditions. Then, we placed all animals in DD, beginning as an extension of the scheduled dark phase. Animals were maintained in DD for 3 weeks. Next, we placed all animals in LL, beginning at the average time of activity onset in shams. Animals were maintained in LL for 3 weeks, and finally placed back in 12:12 LD conditions with lights on at ZT0 until all animals re-entrained (approximately 4 weeks).

One-hour masking pulses were then administered in 3-day cycles, as described previously [24]: day 1 =  maintenance day (12:12 LD); day 2 =  baseline day (12:12 LD); and day 3 =  pulse day. Only one 1-h pulse was given per pulse day. 1-h dark pulses were given during the light phase at ZT0, 1, 6, or 10. (The ZT0 dark “pulse” was the first hour of darkness upon transfer from LD to DD). Following the full set of dark pulses, a 1-h light pulse was given during the dark phase at ZT14. Cage changes and food and water replenishment was only done on the maintenance day. At the end of the experiment, animals were sacrificed for histological analysis.

#### Histology

Animals were euthanized by intraperitoneal injections of sodium pentobarbital (Ovation Pharmaceutical, Deerfield, IL, USA) and immediately perfused transcardially with 0.01 M phosphate-buffered saline (PBS), pH 7.2, followed by 4% paraformaldehyde (Sigma-Aldrich; PFA) with 75 mM lysine (Sigma-Aldrich) and 10 mM sodium periodate (Sigma-Aldrich) in 0.1 M PB (PLP). Following perfusion, brains were immediately removed and post-fixed in PLP for 4-h, then transferred to a 20% sucrose solution (J.T. Baker, Phillipsburg, NJ, USA), and stored at 4°C for at least 48-h. Brains were sectioned in 30 µm coronal sections using a cryostat and organized into three series. One series of brain sections was stained for Nissl using thionin to determine the extent of the lesions. A second series was used for Neuropeptide Y (NPY) immunoreactivity.

#### NPY immunoreactivity

A subset of brains was processed for NPY in sections from the SCN through the IGL (4 shams, 4 complete lesions of the IGL, 1 complete miss of the IGL, and 3 partial lesions of the IGL; see below). The sections were incubated in a rabbit anti-NPY antibody (Peninsula Laboratories, San Carlos, CA, USA; 1∶10,000) at 4°C for 48 h. Sections were then incubated in biotinylated goat anti-rabbit IgG secondary antibody (1:200; Vector Laboratories, Burlingame, CA) followed by amplification using an avidin-biotin peroxidase complex (Vector Laboratories). The NPY-ir was visualized using a diaminobenzidine solution with hydrogen peroxide (Sigma, St. Louis, MO). Sections were mounted onto gelatin-coated glass slides, dehydrated, and coverslipped with dibutyl phthalate xylene (DPX; Sigma-Aldrich). Photomicrographs of the SCN and IGL were taken using a high-resolution digital camera (Carl Zeiss, AxioCam MRC; Göttingen, Germany) connected to a Zeiss light microscope (Axioskop 2 Plus) to examine the loss of NPY in lesioned animals.

To determine the extent of the lesions, thionin stained sections were evaluated independently by two experimenters who were not aware of the behavioral effects of the lesion for the individual animals. Raters traced the extent of the areas that were completely devoid of cells. Independent raters were in complete agreement upon whether or not the IGL was completely lesioned, partially intact, or completely intact. Brain sections with lesions of the IGL and/or surrounding regions were drawn at the rostral, middle, and caudal levels across the IGL using a projection scope (Ken-A-Vision, Model X-1000, Raytown, MO, USA). These drawings were transferred to a computer using a drawing tablet (WACOM, Vancouver, WA) and image software (Adobe Photoshop CS5, Adobe, San Jose, CA, USA). Drawings depicting the area of the lesions were overlaid on those of corresponding brain sections of grass rats, using the Paxinos & Watson rat atlas [28] as a guide for identifying brain areas.

#### Data Analysis

Histological evaluation of the sections identified three animals with either unilateral or partial bilateral damage to the IGL and one with a lesion that spared the IGL completely, with damage primarily to the dorsolateral geniculate nucleus (DLG). These four cases were excluded from the statistical analyses of behavior, which were based on 20 animals with complete IGL lesions and 8 sham-operated controls. Raw general activity data were converted into actograms using the ClockLab Program (Actimetrics, Wilmette, IL), viewed in Microsoft Excel, and analyzed with SPSS.

To evaluate the effects of light and dark pulses, activity counts from the same 1-h interval from the pulse day and preceding baseline day were compared. For 1-h dark pulses, a 3-way ANOVA was used to analyze the data with a 2×2×4 factorial design [surgical condition (sham vs. IGL lesion) x lighting condition (baseline vs. 1-h dark pulse) x time of day (ZT0, 1, 6 and 10); surgical condition as the between-group factor and both lighting condition and time of day as the within-group factors]. Significant interactions were followed by analyses of simple main effects of the pulses within ZTs and surgical condition using paired sample t-tests. For the 1-h light pulse, a 2-way ANOVA was used to analyze the data with a 2×2 factorial design [surgical condition (sham vs. IGL lesion; between-group factor) x pulse (baseline vs. 1-h light pulse; within-group factor)]. A significant interaction was followed by paired t-tests, which were used to analyze the simple main effects of the light pulses within each surgical condition.

In LD conditions, we examined day-night activity by averaging the total activity during the day and night for 5 days for each animal prior to surgery and comparing it to average activity during the day and night, also for 5 days, at 14 days after surgery. We used these data to create an actogram to visualize 1-h activity counts within a 24-h period in animals with complete IGL lesions and in shams. A 2-way ANOVA was used to analyze these data with a 2×2 factorial design [surgical condition (sham vs. IGL lesion; between-group factor) x time of day (day vs. night; within-group factor)] separately prior to surgery and after surgery. In addition, for the postsurgery time period, a 2-way ANOVA was used with a 2×24 factorial design [surgical condition (sham vs. IGL lesion; between-group factor) x ZT; within-group factor)]. A significant interaction was followed by an analysis of the simple main effects of surgical condition within each ZT using independent sample t-tests. This data set in LD conditions was also used to produce day-night ratios by dividing the total average activity during the day by the total average activity at night. For day-night ratios, a 2-way ANOVA was used with a 2×2 factorial design [time of surgery (presurgery vs. postsurgery; within-group factor) x surgical condition (lesion vs. sham; between-group factor)]. Following a significant interaction effect, paired t*-*tests were used to analyze differences between presurgery and postsurgery day-night ratios within each surgical condition. The same LD data were used to calculate the percentage change in activity levels from presurgery to postsurgery during the day and at night for shams and animals with complete IGL lesions. The total amount of activity presurgery was subtracted from the total amount of activity postsurgery, divided by the total amount of activity presurgery, and multiplied by 100. A 2-way ANOVA was used with a 2×2 factorial design [time of day (day vs. night; within-group factor) x surgical condition (lesion vs. sham; between-group factor)]. A significant interaction was followed by analyses of the simple main effects of surgical condition and time of day using independent (surgical condition) or paired (day vs. night) t-tests.

To analyze patterns of activity in DD and LL, we first calculated the period of each animal’s rhythm and divided that by 24 to get the length of a “circadian hour” for that individual. We then averaged the activity counts during each of these 24 intervals for 5 days in DD and LL for each animal. Several animals were excluded from these analyses because the onset and offset of activity was too difficult to detect.

Finally, for all animals, alpha and period were determined in LL and DD conditions. Alpha was defined as the interval from the onset of the early morning bout to the offset of the evening bout. Onset and offset were identified as described previously [29], by using the Clocklab program (Actimetrics, Wilmette, IL; version 2.6.1) in conjunction with visual inspection of double plotted actograms. Two-way ANOVAs were performed separately for the data on alpha and period using 2×2 factorial designs [lighting condition (DD vs. LL; within-group factor) x surgical condition (sham vs. IGL lesion; between-group factor)]. Significant interactions were followed by evaluation of simple main effects using appropriate t-tests.

For all tests, alpha was set at.05. To adjust for multiple comparisons, a Bonferroni correction was applied. All means are presented with their standard errors.

## Results

### Histology

**Figure 1:**
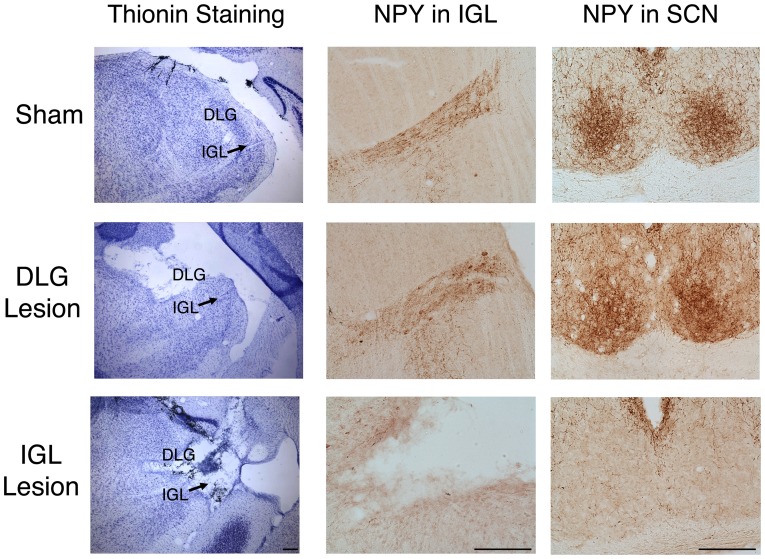
Histology from representative lesioned and sham-operated grass rats. Thionin stained sections through the lateral geniculate (left column) and NPY-labeled sections through the IGL (middle column) and the SCN (right column) from a representative sham (top row), a grass rat with a lesion that missed the IGL but destroyed the DLG (middle row), and from a grass rat with complete IGL lesions (bottom row). NPY was present in the IGL and SCN of shams and grass rats with DLG lesions, whereas NPY was absent in the IGL of grass rats with complete IGL lesions (bottom row). Scale bar represents 400 µm. Abbreviations: DLG: dorsolateral geniculate nucleus; IGL: intergeniculate leaflet; SCN: suprachiasmatic nucleus; NPY: Neuropeptide Y.

**Figure 2:**
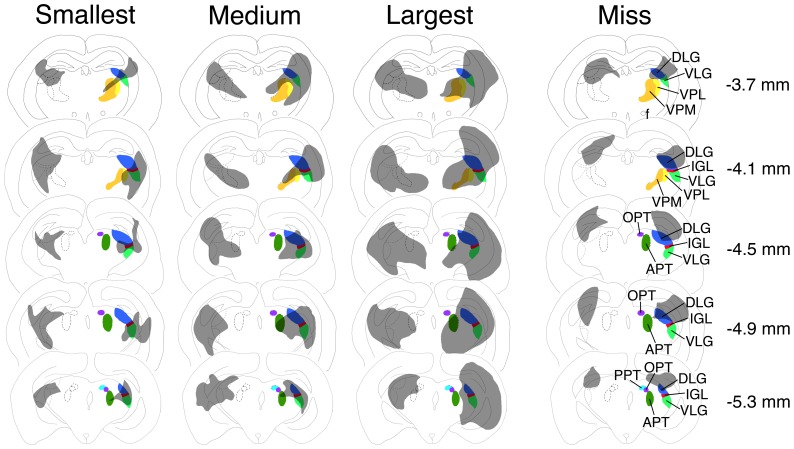
Smallest, medium-sized, and largest electrolytic lesions that destroyed the intergeniculate leaflet bilaterally, along with an animal that had lesions that left the IGL intact (i.e., “miss”), seen in five coronal sections. Millimeters caudal to bregma are indicated on the right. Behavioral data for the smallest IGL lesions and miss are in Figure 3 (labeled IGL Lesion #1 and DLG lesion, respectively). Abbreviations: DLG: dorsolateral geniculate nucleus; IGL: intergeniculate leaflet; VPL: ventral posterolateral nucleus; VPM: ventral posteromedial nucleus; OPT: olivary pretectal nucleus; APT: anterior pretectal nucleus; PPT: posterior pretectal nucleus.

Three groups of animals were identified based on histology: those with (1) complete, bilateral IGL lesions (*n* = 20) (i.e., “hits”; defined as an area devoid of cells in the IGL in thionin-stained sections), (2) unilateral, partial, or no damage in the IGL (*n* = 4) (i.e., “misses”), and (3) shams (*n* = 8) (Figure 1). To confirm the lesion of IGL, brain tissue from a subset of the animals was processed for NPY immunostaining. NPY-ir was seen in both the IGL and the SCN of shams and of those with lesions deemed to be misses. In contrast, in lesioned animals in which the IGL could not be seen in thionin stained material, NPY was absent in the IGL as well as in the SCN, providing further evidence that these lesions completely destroyed the IGL. The brain regions that were damaged following these lesions are depicted in Figure 2. The smallest complete bilateral lesion of the IGL also damaged parts of the dorsolateral geniculate nucleus (DLG). The largest complete bilateral lesion of the IGL also included partial lesions of the DLG, VLG, hippocampus, olivary pretectal nucleus (OPT), and the thalamus [including the ventral posterolateral thalamus (VPL) and ventral posteromedial thalamus (VPM)]. Lesions that were deemed as misses had damage to the DLG, VLG, hippocampus, and/or parts of the IGL, but in all of these cases, at least some IGL tissue remained intact.

### In 12:12 LD conditions, complete IGL lesions resulted in a night-active profile

**Figure 3:**
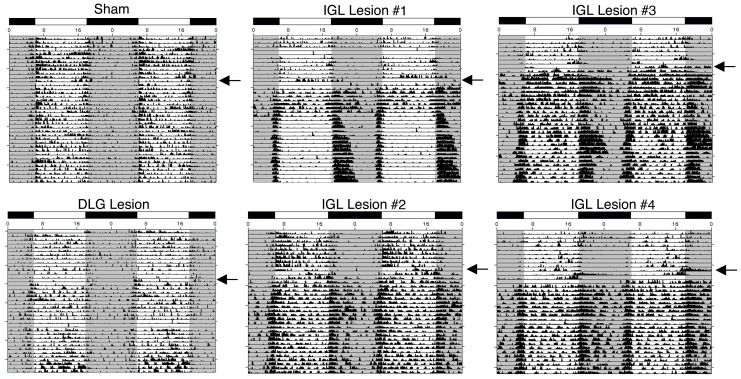
Effects of lesions on behavior before and after surgery in LD conditions. Actograms depicting general activity before and after surgery in representative animals with sham lesions (top left), a miss (DLG lesion, bottom left), and complete IGL lesions (records from 4 such animals are shown in the middle and right columns). Lesion size for the representative DLG lesion (i.e., miss) and IGL Lesion #1 are presented in Figure 2. Day of surgery is indicated by the black arrow. White/black bar indicates the 12:12 hour light/dark cycle. Note the lack of change in activity patterns in the sham and DLG lesion following surgery. In contrast, there is a significant increase in nighttime activity in IGL lesioned animals following surgery.

**Figure 4:**
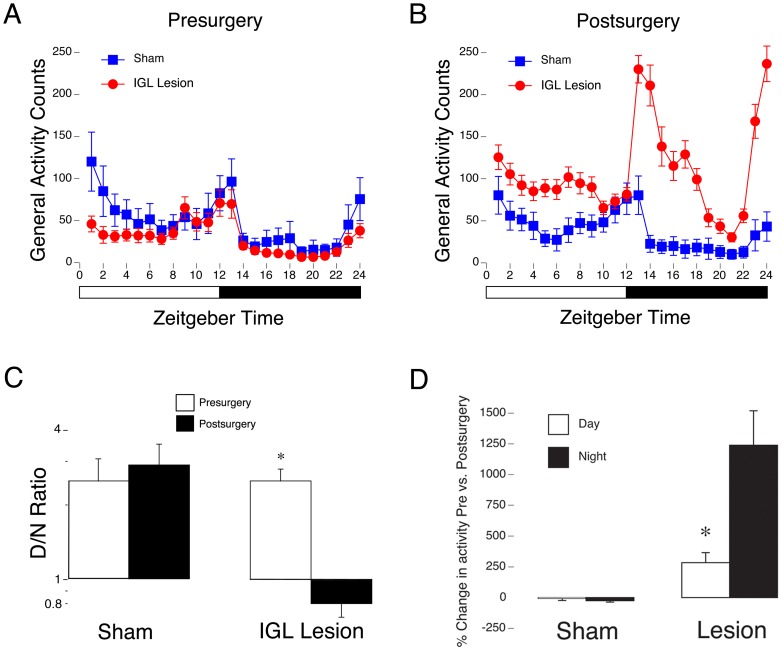
Quantitative analyses of behavior in LD conditions before and after surgery. 5-day average patterns of activity in LD across the day and night in shams (blue squares) and animals with complete IGL lesions (red circles); behavior from prior to surgery is shown in (**A**), and after surgery in (**B**). White/black bar indicates the 12:12 hour light/dark cycle. Prior to surgery all animals were more active during the light phase than the dark phase. Following surgery, shams remained more active during the day, whereas animals with complete IGL lesions became more night-active. (**C**) Day/night (D/N) ratios of sham animals and of animals with complete IGL lesions prior to surgery and following surgery. Shams exhibited no change in D/N ratios following surgery, whereas grass rats with IGL lesions exhibited a significant decrease in D/N ratio from presurgery to postsurgery. *  =  significant difference from presurgery to postsurgery. Means + SE. (**D**) Percent change in activity levels from presurgery to postsurgery for shams and animals with IGL lesions during the day and at night. Note the significant increase in nighttime activity in lesioned animals as compared to the day. *  =  significant difference between day and night.

Prior to surgery in 12:12 LD conditions, all animals displayed robust daily activity patterns, with general activity most concentrated during the light phase, as previously reported for grass rats [24], [30] (Figures 3, 4A, 4C). Figure 3 depicts actograms from six individual animals in 12:12 LD prior to and following surgeries. Records are from one sham-operated animal, one animal with a lesion that spared the IGL (i.e., DLG lesion), and four animals with complete bilateral IGL lesions (labeled #1 - #4). Animals with sham lesions, unilateral or partial lesions of the IGL (not shown), and complete IGL misses (i.e., DLG lesion; area of lesion is depicted in Figure 2) exhibited a post-surgical day-active pattern. In sharp contrast, lesions that destroyed the IGL resulted in a night-active pattern of general activity. IGL Lesion #1, which had the smallest complete lesion (area of the lesion is depicted in Figure 2), exhibited an increase in nocturnal activity with some reduction in activity during the day. Animals with larger lesions that also included all of the IGL showed post-surgical patterns similar to those of lesioned animals # 2 – 4 (Figure 3). These animals also became predominantly night-active after surgery, but they also displayed heightened activity during the day.

Prior to surgery (Figure 4A), the temporal pattern of daily activity was not different between animals in the sham and lesion group (the ANOVA revealed no significant effect of surgical condition: F_1,26_ = 1.6, *p* = .211, and no significant interaction between time of day and surgical condition: F_1,26_ = 0.3, *p* = .579). Following surgery (Figure 4B), in LD conditions, the ANOVA revealed a significant interaction between time of day and surgical condition (F_1,26_ = 8.7, *p* = .007). The ANOVA used to probe the generality of this effect of surgery across ZTs revealed a significant interaction between ZT (i.e., ZT 1 – 24) and surgical condition (i.e., sham vs. lesion) (F_23,598_ = 6.4, *p*<.001). Analysis of the simple main effects of surgery at each ZT showed that at all time points at night, except ZT19, ZT20, and ZT21 (t_26_s<2.1, *p*s>.042), general activity counts were significantly higher for animals with IGL lesions as compared to those with sham lesions (t_26_s>3.4, *p*s ≤.002). The only time point during the day at which general activity was significantly different between groups was ZT5 (t_26_ = 3.5, *p* = .002).

Day/Night (D/N) ratios prior to and post-surgery were analyzed in sham and lesioned animals (Figure 4C). Prior to surgery, animals were approximately 2.5 times more active during the day as compared to the night. After surgery, there was a significant decrease in D/N ratio only in lesioned animals. The ANOVA revealed a significant pre/post surgery time x surgical condition interaction (F_1,26_ = 14.2, *p* = .001). Simple main effects of pre/post time of surgery revealed a significant decrease in D/N ratio from presurgery to postsurgery for lesioned animals (t_19_ = 5.1, *p*<.001), but not for shams (t_7_ = 1.3, *p* = .225).

For percentage change in activity from presurgery to postsurgery (Figure 4D), the ANOVA revealed a significant surgical condition x time of day (day or night) interaction (F_1,26_ = 5.1, *p* = .032). Analysis of the simple effects of surgical condition found significant differences for both day (t_26_ = 2.3, *p* = .032) and night (t_26_ = 2.8, *p* = .009). The simple main effect of time of day was not significant for shams (t_7_ = 1.5, *p* = .167), but it was significant for the lesion group (t_19_ = 3.6, p = .002); as shown in Figure 4D, although overall post-surgical activity was increased in the lesioned animals, this increase was approximately five times larger at night than during the day.

### In constant conditions, complete IGL lesions resulted in changes in alpha, but not period

**Figure 5:**
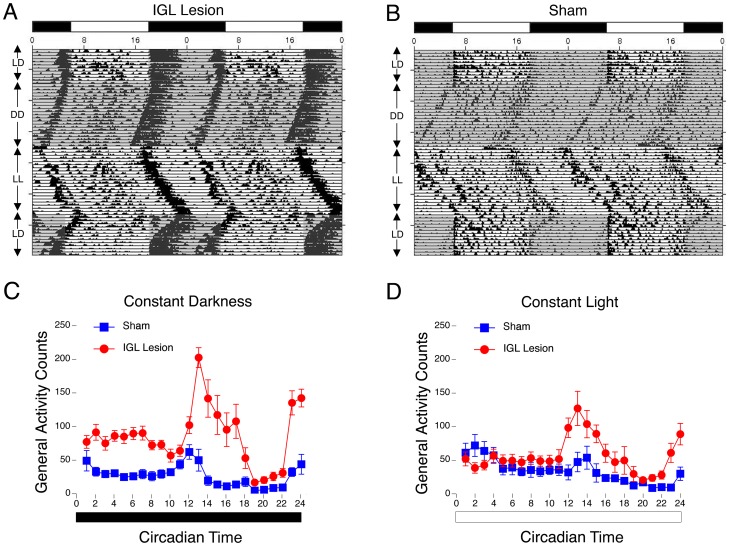
Effects of lesions on behavior in constant conditions. Double-plotted actograms from 1 representative animal with complete IGL lesions (**A**) and 1 representative sham-operated animal (**B**) during periods of LD, DD, LL, and LD. Note the increase in activity at night in animals with IGL lesions, which persists and free-runs in constant conditions. (**C**) 5-day average activity patterns of sham-operated animals (Blue squares) and animals with complete IGL lesions (Red circles) kept in DD and in LL (**D**). Note that although constant light suppresses activity in IGL lesioned animals, the same overall pattern of activity is observed.

Figures 5A and 5B present actograms of a representative animal with bilateral IGL lesions and a representative sham following surgery in LD, DD, LL, and finally LD. For alpha, the ANOVA revealed a significant interaction between lighting condition (i.e., DD vs. LL) and surgical condition (F_1,26_ = 86.9, *p*<.001). Animals with IGL lesions had an alpha in DD of 16.55±0.30 hours, which was significantly longer (t_26_ = 6.4, *p*<.001) than that of shams (13.02±0.45 hours; see Table 1). In contrast, in LL, animals with IGL lesions had an alpha of 14.19±0.30 hours, which was significantly shorter (t_26_ = 4.5, *p*<.01) than that for shams (16.58±0.37 hours; see Table 1). Further, animals with IGL lesions exhibited a significantly decreased alpha from DD to LL (see Table 1; t_19_ = 7.8, *p*<.001), whereas shams significantly increased their alpha from DD to LL (see Table 1; t_7_ = 5.3, *p* = .001).

**Table 1:** Means (SE) for period and alpha in hours during LD, DD and LL conditions for shams and grass rats with IGL lesions.

	LD (Presurgery)	LD (Postsurgery)	DD		LL	
	Alpha	Alpha	Period	Alpha	Period	Alpha
Sham	13.75 (0.39)	13.86 (0.41)	23.73 (0.03)*	13.02 (0.45)*	24.41 (0.04)	**16.58 (0.37)**
Lesion	13.45 (0.16)	15.83 (0.21)	23.80 (0.02)*	16.55 (0.30)*	24.49 (0.05)	**14.19 (0.30)**

Bolded values are significantly different between groups (i.e., sham vs. lesion). * indicates a significant difference within groups (i.e., DD Period vs. LL Period, DD Alpha vs. LL Alpha).

For period, the ANOVA revealed a significant main effect of lighting condition (i.e., DD vs. LL; F_1,26_ = 216.9, *p*<.001), but not of surgical condition (i.e., sham vs. lesion; F_1,26_ = 2.5, *p* = .123), and no significant interaction between these two variables (F_1,26_ = .05, *p* = .828). The simple main effects of lighting condition were significant such that both animals with IGL lesions (t_19_ = 12.4, *p*<.001) and shams (t_7_ = 17.6, *p*<.001) exhibited a longer period in LL than DD.

The post-surgical general activity patterns displayed in LD persisted in DD and LL (see Figures 5C and 5D), indicating that the increased nocturnal activity of animals with IGL lesions was not a passive response to the LD cycle. When animals were transferred back to LD from LL it took several days of transients before rhythms became entrained (Figure 5A).

### Dark pulses increased general activity in animals with complete IGL lesions but not in shams

**Figure 6:**
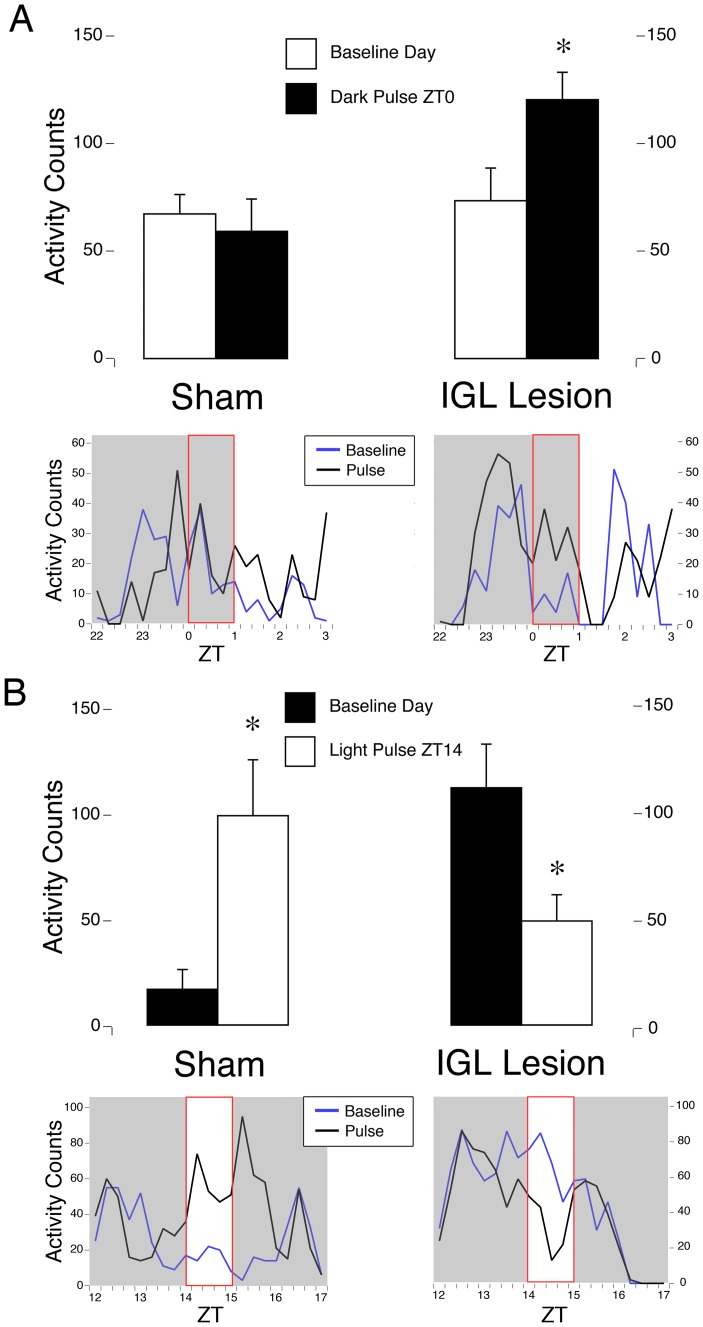
Masking responses to light and dark pulses. (**A**) Masking response to a dark pulse in LD conditions during the light phase at ZT0. In shams, activity was unchanged in response to dark pulses at ZT0. In animals with complete IGL lesions, dark pulses resulted in a significant increase in activity at ZT0. *  =  significant difference between baseline day (White bars) vs. 1-h dark pulse (Black bars). Means + SE. Representative activity patterns for 2-h prior to, 1-h during, and 2-h following the dark pulse are presented for one sham (left) and one IGL lesioned animal (right) during a baseline day (blue line) and on a day when dark pulse was presented at ZT0 (black line). (**B**) Response to a light pulse in LD during the dark phase at ZT14. In shams, activity was significantly increased in response to a light pulse at ZT14. In animals with IGL lesions, light pulses resulted in a significant decrease in activity at ZT14. *  =  significant difference between baseline day (Black bars) vs. 1-h light pulse (White bars). Means + SE. Representative activity patterns for 2-h prior to, 1-h during, and 2-h following the light pulse are presented for one sham (left) and one IGL lesioned animal (right) during a baseline day (blue line) and on a day when light pulse was presented at ZT14 (black line).

For animals that were presented with 1-h pulses of darkness, a 3-way interaction between time of dark pulse, surgical condition and lighting condition was not significant (F_3,26_ = 0.4, *p* = .736). The 2-way lighting condition x surgical condition interaction was significant (F_1,26_ = 5.7, *p* = .024), but the time of dark pulse x surgical condition (F_3,26_ = 1.5, *p* = .212), and the time of dark pulse x lighting condition interactions were not (F_3,26_ = 0.5, *p* = .651). Evaluation of the simple effect of lighting condition revealed a significant increase in activity in response to darkness only at ZT0 (t_19_ = 4.4, *p*<.001; see Figure 6A), with a near significant effect at ZT1 (t_19_ = 2.5, *p* = .023; alpha set at.0125 following a Bonferroni correction) for lesioned animals. Dark pulses had no effect on shams at any time point (t_7_s<0.9, *p*s>.398). See Table 2 for a summary of the effects of dark pulses presented at ZT1, ZT6, and ZT10.

**Table 2:** Means (SE) for 1-h dark pulses presented at ZT1, ZT6, and ZT10 in shams and grass rats with IGL lesions.

	ZT1	ZT6	ZT10
**Sham**			
Baseline	49.4 (21.9)	35.6 (11.5)	43.0 (14.8)
Dark Pulse	59.1 (25.1)	32.6 (11.1)	28.8 (8.2)
p-value	.791	.788	.398
**Lesion**			
Baseline	36.5 (6.9)	52.1 (39.7)	24.8 (9.8)
Dark Pulse	67.2 (11.0)	74.9 (57.6)	43.4 (11.4)
p-value	.023	.169	.135

P-values are also presented for analyses of simple main effects of dark pulses within ZTs and surgical condition.

### A light pulse decreased general activity in animals with complete IGL lesions and increased it in control animals

For animals that were presented with a light pulse at ZT14, the ANOVA revealed a significant interaction between lighting condition (i.e., light pulse vs. baseline day) and surgical condition (i.e., lesion vs. sham) (F_1,26_  = 17.3, *p*<.001). Evaluation of the simple effect of lighting condition revealed a significant difference at ZT14 in both lesioned (t_19_ = 3.0, *p* = .007) and sham (t_7_ = 4.8, *p* = .002) animals, but critically, these were in opposite directions. That is, a 1-h light pulse at ZT14 *increased* general activity in shams, whereas it *decreased* general activity in animals with IGL lesions (see Figure 6B).

## Discussion

Nile grass rats are diurnal in their natural habitat [31], and their general activity in the lab is heavily concentrated during the light phase of a 12:12 LD cycle [24], [32]. The present findings reveal that the maintenance of diurnality in these animals is dependent upon the IGL. Specifically, complete IGL lesions increased activity in a phase-dependent manner such that the overall day-night activity ratio changed from 2.5 before the surgeries to 0.8 after them. Importantly, this change did not reflect a simple reversal in the activity profile. Lesions actually increased activity at one point during the light phase (ZT5) as well as across much of the dark phase, but they had no effect at all during most of the of the light phase (i.e. 11 of the 12 hour intervals), or during a 3 hour period of the second half of the dark phase. Although the pattern has clear crepuscular elements, activity remained well over four times higher in lesioned than control animals across the first six hours of the dark phase; this portion of the activity rhythm of our lesioned animals is very similar to those seen in intact mice [33] and hamsters [34]. Taken together, these patterns suggest that the IGL is essential for the maintenance of species-typical patterns of diurnality in these animals, but that its role is a complex one and its influence may change across the day. There are likely to be multiple mechanisms that support or inhibit activity at particular phases of the circadian cycle. Phase-dependent changes in activity have been induced in nocturnal rodents in a variety of ways, such as via genetic manipulations that render mice deficient in the SCN output signal prokineticin 2 [35]. The present data suggest that the IGL of grass rats might normally contribute to diurnality by counteracting some processes that would otherwise drive activity up in a time- and light-dependent manner.

IGL lesions also induced a reversal in masking responses to photic stimuli. In direct tests of masking, light stimulated activity at ZT14 in intact grass rats (i.e., positive masking), but suppressed it in lesioned ones (i.e., negative masking). Interestingly, in nocturnal hamsters, IGL lesions increase the suppression of activity by light [6], thus suggesting that one function of the IGL may be to decrease the suppressive effect of light on activity, regardless of the chronotype of the species. Our results also support a role for the IGL in suppressing activity in response to dark pulses. Masking responses to dark pulses are normally absent in grass rats [24], but emerge after IGL lesions; these responses to darkness were only observed during the early part of the day, suggesting that the effects of the lesions on positive masking by darkness are phase-specific. Importantly, our grass rats with IGL lesions responded to acute presentations of light and darkness in similar ways to intact nocturnal species; both increase their activity in response to dark pulses and reduce activity in response to light pulses [1], [24]. The effect of IGL lesions on rhythms in constant light and constant darkness also supports a role for the IGL in modulating masking. Specifically, the expansion and compression of alpha in lesioned animals in DD and LL, respectively, was exactly the opposite of what we saw in the control animals. In addition, IGL lesioned animals’ total activity was suppressed by constant light, but their overall pattern of circadian activity did not change. These effects of IGL lesions may generalize to other diurnal species, since in diurnal degus, IGL lesions also result in an expansion of alpha [19]. Our results support the hypothesis that the IGL plays in an important role in mediating masking to photic stimuli in grass rats.

Altogether, our results suggest that the contribution of the IGL to the species-typical expression of activity patterns in grass rats is driven by its influence on both masking and circadian mechanisms. The role of the circadian system in generating an enhanced night-active pattern in lesioned animals is apparent from the fact that the post-surgical patterns were unaffected by a transfer from LD to DD. Theoretically, the change in distribution of activity caused by IGL lesions could involve a change in the coupling between the LD cycle and the SCN. This is unlikely since the phase of the oscillator of the SCN is constant across diurnal and nocturnal species [36] and across different chronotypes within species [37]. Further, we found no evidence that the influence of light on the period of the rhythm in constant conditions was affected by IGL lesions. Therefore, we hypothesize that the IGL has a substantial influence on the pattern of coupling between the SCN and activity, but that it plays only a minor role, if any, in the coupling of the oscillator to the LD cycle. However, coupling of an endogenous oscillator to a LD cycle is mediated not only through the effects of light on the period (parametric effects), but also on the phase (nonparametric effects) of that oscillator [38]. We did not examine nonparametric effects of light on the oscillator in this study, or how those effects may be altered by IGL lesions. A comparison of light phase response curves of grass rats before and after IGL lesions is needed to evaluate this question further.

Lesions that affected masking responses and nighttime activity included damage to more than the IGL, but lesions of comparable size that spared the IGL either partially or completely had no apparent effects on those measures. Thus, the most parsimonious interpretation of our data is that the IGL is the critical structure for the effects we observed. However, an alternative interpretation is that the increase in nighttime activity and changes in masking emerge as a result of concurrent damage to the IGL and to nearby regions within the lateral geniculate complex [39]. More discrete lesions of the IGL are needed to rule out this alternative explanation. Since degus with relatively small lesions that included the IGL do not exhibit a significant enhancement of activity at night [19], it is possible that large lesions are necessary in order for this effect to be observed in diurnal species. Alternatively, the difference between what we observed in grass rats and what has been reported in degus may reflect a species difference with respect to the magnitude of the influence that the IGL has on circuits that control activity profile, or perhaps more fundamental features of its role in regulation of rhythms.

Although grass rats display robust diurnal profiles in their natural habitat [31] and under most conditions in the lab [24], [32], when given access to wheels, a subset of them adopts wheel running patterns that bear a striking resemblance to patterns of general activity seen here in lesioned animals during the dark phase of a 12:12 LD cycle [40]. In those intact but night-active grass rats, the basic nocturnal profile persists in DD [41], and acute effects of light and dark pulses are the same as those seen here in IGL-lesioned animals (Langel, unpublished data). Specifically, in night-active wheel-runners, light pulses at all time points, except ZT20, result in negative masking, whereas dark pulses only result in positive masking during the early part of the day. Therefore, in animals that become more active at night (i.e., wheel-runners and IGL lesioned animals), masking responses are aberrant in a phase-specific fashion. In addition to their similarities in activity profiles and masking responses to light and darkness, both night-active wheel-runners and grass rats with IGL lesions exhibit significantly increased total amounts of activity associated with the switch in chronotype [40]. These similarities suggest that neural mechanisms shaping the temporal structure of activity/rest patterns across the night could be similar in the two contexts.

In summary, we have shown here that lesions that result in complete destruction of the retinorecipient IGL produce an increase in nighttime activity, and reverse the masking responses to light and darkness with respect to the normal profile of the species. This discovery raises many questions about how the IGL and surrounding regions work to promote species-typical diurnal patterns in intact grass rats, and about how its influence on two very different contributors to diurnality, masking and circadian regulation, is linked. The data also raise interesting questions about relationships between IGL outputs that appear to play a role in damping activity in a time- and light-dependent fashion, and other systems that may do the opposite, and how these differ in day and night-active animals. In nocturnal rodents the IGL is known to project to approximately 100 brain regions [42]–[45], many of which play important roles in regulation of sleep and wakefulness. The next question is where and how might the IGL influence activity within these circuits.
